# Tennis shot side-view and top-view data set for player analysis in Tennist

**DOI:** 10.1016/j.dib.2024.110438

**Published:** 2024-04-18

**Authors:** Kalin Guanlun Lai, Hsu-Chun Huang, Wei-Ting Lin, Shang-Yi Lin, Kawuu Weicheng Lin

**Affiliations:** a Department of Computer Science & Information Engineering, National Kaohsiung University of Science and Technology, Kaohsiung, Taiwan; b Department of Mechanical Engineering, National Kaohsiung University of Science and Technology, Kaohsiung, Taiwan; c Physical Education Office, National Kaohsiung University of Science and Technology, Kaohsiung, Taiwan

**Keywords:** Object tracking, Physical simulation, Tennis ball flying, Sports technology

## Abstract

Tennis is a popular sport, and the introduction of technology has allowed players to diversify their training. Tennis ball tracking is currently a focal point, serving not only to assist referees but also to enhance sports analysis. We introduce the Tennis Shot Side-View and Top-View Dataset, which serves as an invaluable resource for analyzing tennis movements and verifying landing positions after flight. This dataset combines side-view and top-view video clips, capturing various shot types and player movements from both outdoor and indoor fields. The dataset includes the actual ball positions of each clip for verification purposes. The Tennis Shot Side-View and Top-View Dataset represents a significant advancement in tennis research. Its multidimensional nature opens doors for in-depth player analysis, performance enhancement, and strategy development. We believe that this dataset will be a valuable asset to the tennis community, fostering innovation and excellence in the sport.

Specifications Table

**Table:** 

Subject	Computer Science / Computer Vision and Pattern Recognition
Specific subject area	Image Processing; 3D Reconstruction; Object Tracking in Video; Tennis Tracking; 3D Ball Trajectory Simulation
Data format	Raw
Type of data	Video file using H.264/MPEG-4 AVC codec (.mp4) .csv file (the records of real landing point)
Data collection	The dataset includes 472 clips, organized to two main directories: “Outdoor Field” and “Indoor Field”. These directories are divided into sub-directories, “Straight Shot” and “Cross-court Shot”. Both of shots (tennis forehand shots) directories are split into two view folders, “Top-View” and “Side-View”: • “Outdoor Field/Straight Shot/Top-View” includes 18 clips representing the top-view of tennis straight shot in outdoor field. • “Outdoor Field/Straight Shot /Side-View” includes 18 clips representing the side-view of tennis straight shot in outdoor field. • “Outdoor Field/Cross-court Shot/Top-View” includes 20 clips representing the top-view of tennis cross-court shot in outdoor field. • “Outdoor Field/Cross-court Shot/Side-View” includes 20 clips representing the side-view of tennis cross-court shot in outdoor field. • “Indoor Field/Straight Shot/Top-View” includes 99 clips representing the top-view of tennis straight shot in Indoor field. • “Indoor Field/Straight Shot/Side-View” includes 99 clips representing the side-view of tennis straight shot in Indoor field. • “Indoor Field/Cross-court Shot/Top-View” includes 99 clips representing the top-view of tennis cross-court shot in Indoor field. • “Indoor Field/Cross-court Shot/Side-View” includes 99 clips representing the side-view of tennis cross-court shot in Indoor field. The CSV files under root folder contain each ball's real landing point coordinates records. The file's name indicates the data is from where: field categories and shot types.
Data source location	National Kaohsiung University of Science and Technology (NKUST). Outdoor field:Tennis Courts in NKUST Indoor field:Stadiums in NKUST
Data accessibility	Repository name: Tennis Shot Side-View and Top-View Data Set for Player Analysis in Tennis Data identification number: 10.17632/75m8vz7jr2.2 Direct URL to data: https://data.mendeley.com/datasets/75m8vz7jr2

## 1 Value of the Data

• Advancing sports technology, our dataset represents a significant contribution to the field of sports technology. Demonstrates how the integration of computer vision technology can be utilized in tennis practice assistance and data analysis.
• Facilitating image recognition in sports, our dataset includes valuable insights into image recognition techniques applied in sports. The accuracy of the data helps researchers and practitioners in the development of recreational games.
• To predict the landing position of the tennis ball after flight, our dataset contains data related to the landing position of the tennis ball after flight. By analyzing the verification of the correct position of the tennis ball after flight, we help to understand ball physics and help to predict the flight pattern of the ball in a real tennis match.
• It is necessary to establish a diversified database of sports data, and through the data collection in different tennis courts, we can understand the difference between the data collection in Outdoor field and Indoor field. It will be helpful for researchers to have a reference on the diversity of data collection.

## 2 Background

Sports-related datasets offer valuable insights into various aspects such as policy-making, consumer decision-making, and sports science. These datasets play a crucial role in monitoring athlete health, managing sports injuries, analyzing athlete skills, and enhancing science and technology [1], [2], [3]. As technology continues to advance, its application in sports-related domains becomes increasingly prevalent. Whether it involves data collection through technology or the utilization of data to develop technological systems, this approach represents the current mainstream trend [4], [5], [6].

In our specific context, our focus lies on the development of interactive entertainment. This entails harnessing computer vision methodologies, including moving object segmentation and color segmentation. Within the realm of sports science and technology, the dataset under consideration serves as a vital resource for research endeavors aiming to uncover the intricate dynamics of tennis gameplay [7,8]. The dataset comprises rich top-view and side-view video clips, facilitating the estimation of the tennis ball's landing position [9].

This dataset offers spatial data from two perspectives, contributing to the establishment of comprehensive landing coordinates for tennis trajectories [10,11]. By integrating computer vision technology, players can predict the landing position of the tennis ball after contact. Our goal with this dataset is to foster the advancement of computer vision applications in tennis-related fields, particularly in interactive entertainment scenarios. The dataset is tailored for validation purposes to ensure its utility and effectiveness across various applications in the field.

## 3 Data Description

Our dataset represents a valuable resource for researchers and enthusiasts in the realm of sports science, computer vision, and tennis analysis [12,13]. We have constructed a unique dataset that captures tennis activities in diverse environments, encompassing both outdoor fields, exemplified by tennis clay courts, and Indoor fields built within stadiums. Indoor fields serve as a valuable resource for players seeking indoor practice environments, a feature less popular in regions such as Taiwan compared to Europe and the United States. Moreover, these Indoor fields in school gymnasiums address the challenge of playing tennis outdoors during adverse weather conditions, facilitating year-round training.

**Fig. 1:**
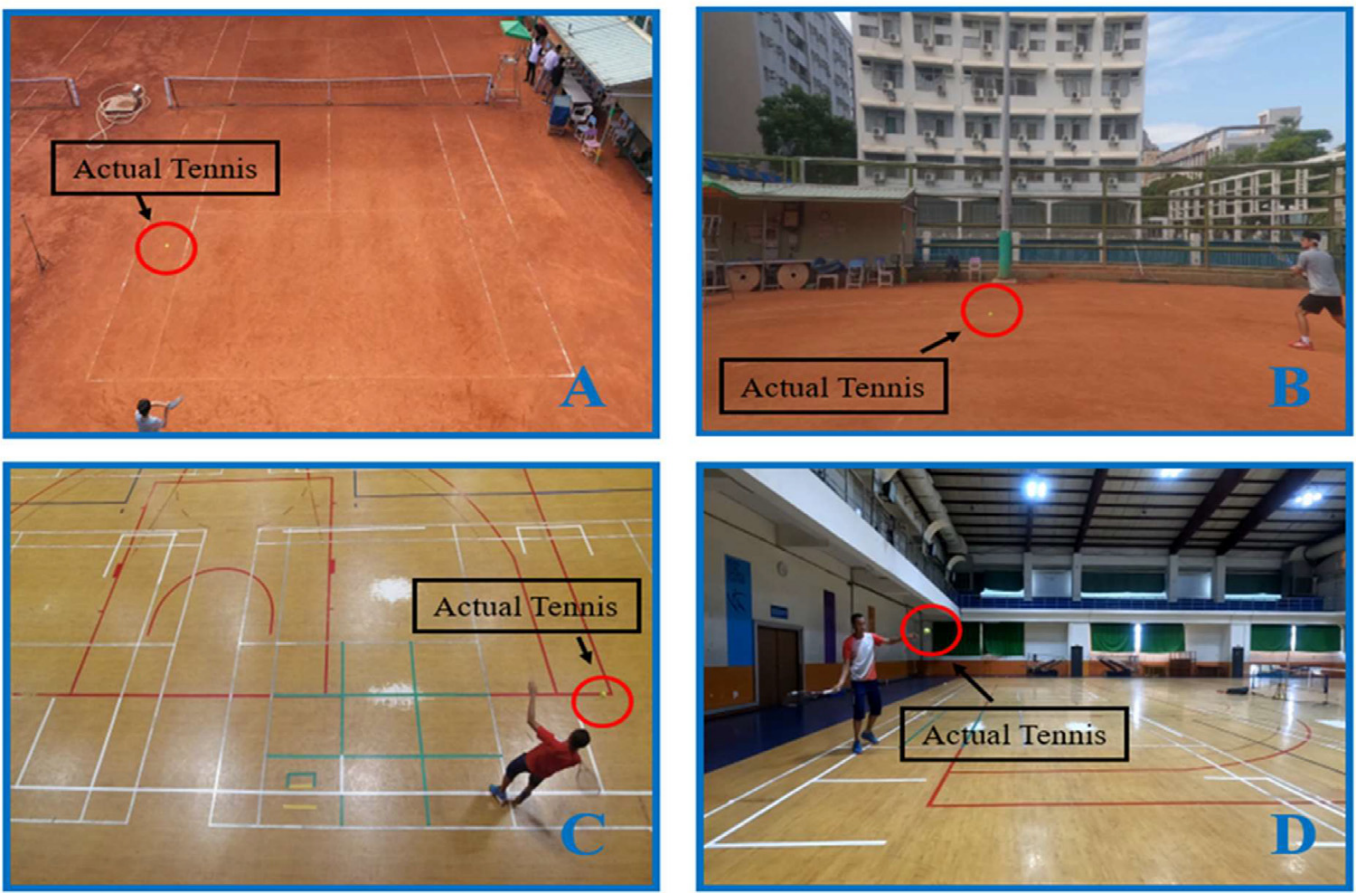
The comparison between the outdoor field and the indoor field is illustrated. (A) Outdoor field top view. (B) Outdoor field side view. (C) Indoor field top view. (D) Indoor field side view.

This experiment was conducted at NKUST, involving both tennis courts and stadiums. Two cameras were utilized for the experiment, and the same participant conducted the experiment in different venues. The data in our dataset is collected from both an outdoor field and an indoor field, which enhances the reliability of our data [14]. In the outdoor field, we specifically chose a clay tennis court because it provides greater accuracy in measuring real distances, as it retains the imprint of the tennis ball on the ground. For the indoor field, we utilize the gymnasium to set up a tennis court, which can be used for data acquisition in the indoor space. The comparison between the outdoor field and the indoor field is illustrated in Fig. 1.

Researchers can track the precise movement trajectories of athletes and the landing positions of tennis balls in dynamic real-world environments. This capability opens avenues for in-depth spatial analysis, enabling investigations into factors such as shot accuracy, player positioning, and the influence of court geometry on gameplay. This dataset contains two parts: 1. clips of players hitting the ball, and 2. the landing position of the ball after the flight (the landing position of each ball).

## 4 Clips Information

The dataset described in this article contains 472 clips of a tennis ball being swung. The data is categorized into two types based on the field: Outdoor field and Indoor field. Within these categories, there are two types of shots: Straight shot and Cross-court shot. Each data item includes Top view clips and side view clips. Researchers can utilize various computer vision and image processing algorithms to recognize and detect these different versions of the dataset. Clips from 2 viewpoints to verify the landing position of a flying tennis ball, revealing the complex dynamics of this fast-paced sport [15,16]. All clips were captured on the campus of the National Kaohsiung University of Science and Technology (NKUST) using a GoPro HERO 10 Black camera, with support from sports research experts and graduate students. Aerial camera operations were employed for top-view imaging. All clips have a resolution of 1080P, with dimensions of 1920 pixels width and 1080 pixels height, and a frame rate of 60 fps. The data collection process is outlined in Table 1.

**Table 1:** Brief description about the data collection.

No.	Particulars	Description
1	Data type	Outdoor field and Indoor field
2	Data categories	Straight shot and Cross-court shot
3	Data item	Top view clips and Side view clips
4	Data format	Video file using H.264/MPEG-4 AVC codec (.mp4) Resolution: 1080P (1920 × 1080 pixels) Frame Rate: 60 fps
5	Period and Date	Outdoor field:March 1, 2023, 10 a.m.-12 p.m. Indoor field:March 2, 2023, 10 a.m.-12 p.m.
6	Participants	The Students of NKUST Tennis Team
7	Location	Outdoor field:Tennis Courts in NKUST Indoor field:Stadiums in NKUST

Source: Author's own organization

## 5 Dataset Information

**Fig. 2:**
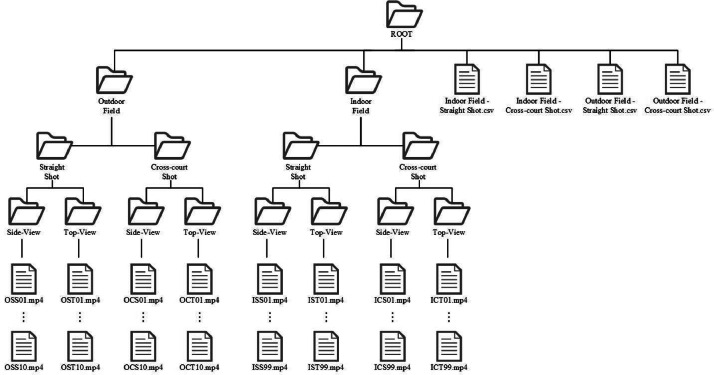
Folder structure.

Because researchers collected data at outdoor field and indoor field, the dataset organized to two main directories. Under these directories, both are divided into two subfolders by shot type (straight shot and cross-court shot). Then, each ball includes two views, side-view and top view, so splitting into two folders in these subfolders. The files in the dataset are named by researchers, following a specific convention. Researchers extract the first letter of the parent folders and assign sequential numbering. For instance, clips within the “Outdoor Field/Straight Shot/Top-View” subfolder are denoted by OST followed by a numerical sequence (e.g., OST01, OST02, ..., OST99). Folder structure is shown in Fig. 2.

**Fig. 3:**
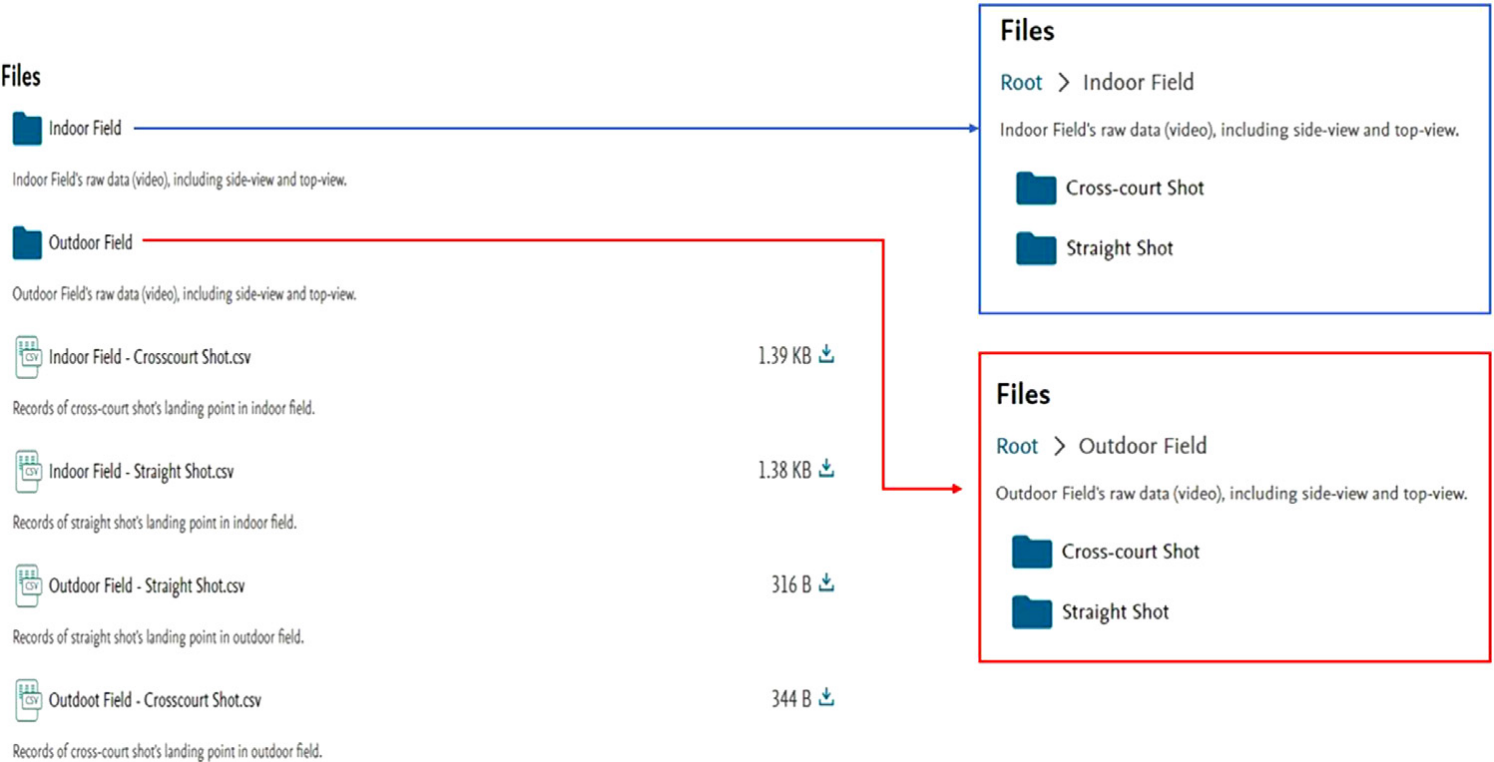
Dataset files on Mendeley Data

The dataset contains 472 clips (both of side-view and top-view are 236 clips), and 4 records files, and it on Mendeley Data shown in Fig. 3 (size on disk is about 12.42 GB (2,601,105,575 bytes)).

Landing Position after Tennis Ball Flying, researchers record each ball's real landing point coordinates. These data are recorded in the CSV files, and the CSV files are under the root folder. The file's name indicates where the data is from: field categories and shot types. An essential aspect of the image acquisition process is the need for actual coordinates. Throughout the data collection process, we measure and record the real landing point's coordinates of the ball each time a participant hits it, using the distance of the closest doubles sideline and the distance of the baseline to present (outside ball represent by negative number). These actual coordinates are crucial for validating the projections. Data category description is provided in Table 2.

**Table 2:** Data category description and capture techniques.

Categories	Description
Straight shot	Total 234 clips, including Outdoor field's Top-view 18 clips, Side-view 18 clips; Indoor field's Top-view 99 clips, Side-view 99 clips.
Cross-court shot	Total 238 clips, including Outdoor field's Top-view 20 clips, Side-view 20 clips; Indoor field's Top-view 99 clips, Side-view 99 clips.

Source: Author's own organization

## 6 Experimental Design, Materials and Methods

The camera setup comprises two parts: top view and side view. For the top view, we positioned the camera directly behind the field, about 1.21 m from the bottom line, and at a height of 5.15 m. For the side view, we set up on the right side of the field, about 2.45 m from the sideline and about 2.40 m from the baseline, with the camera at a height of 1.27 m.

**Fig. 4:**
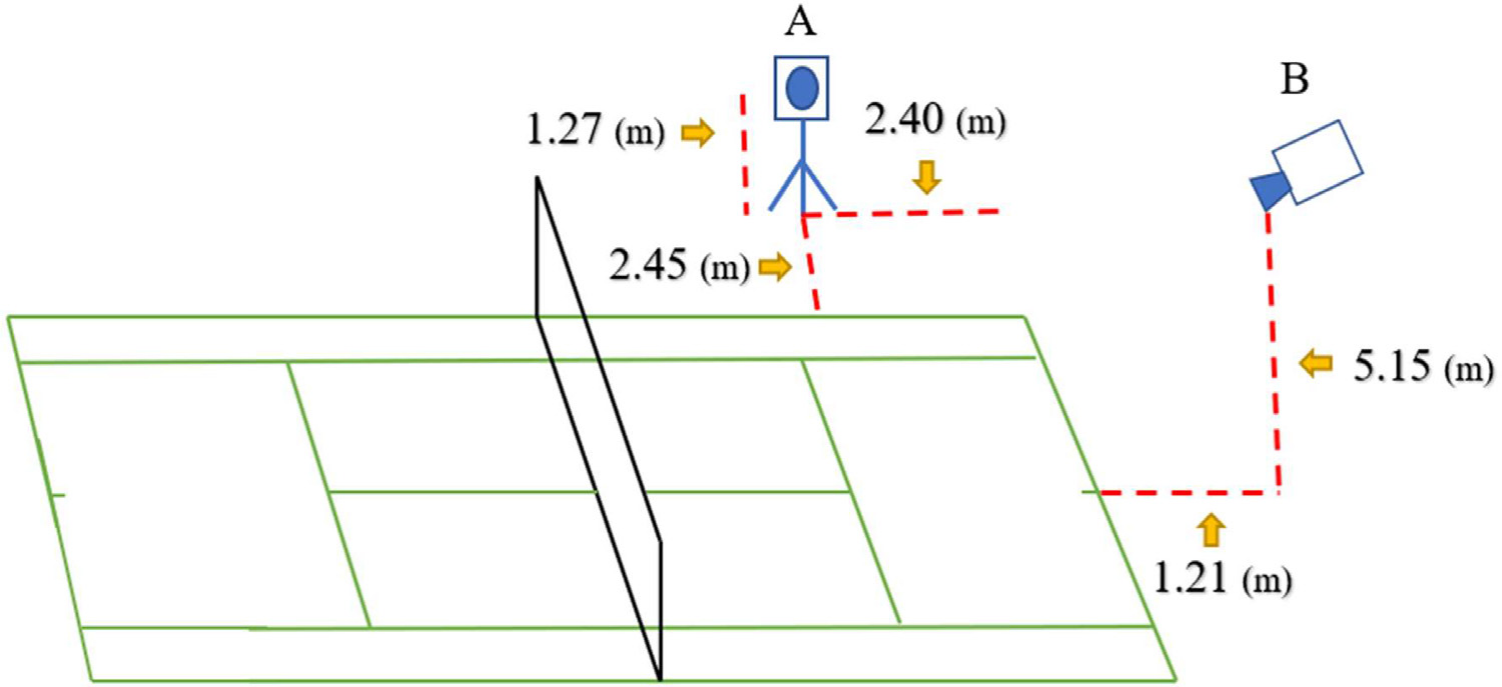
Location map of the camera setup in the experimental field. (A) Top view camera. (B) Side view camera.

Setting up the top view for the Outdoor field proved more challenging, so we utilized an aerial camera to assist in this part. This approach ensured the consistency of the data obtained from both the outdoor field and the indoor field. The location of the camera setup is shown in Fig. 4.

**Fig. 5:**
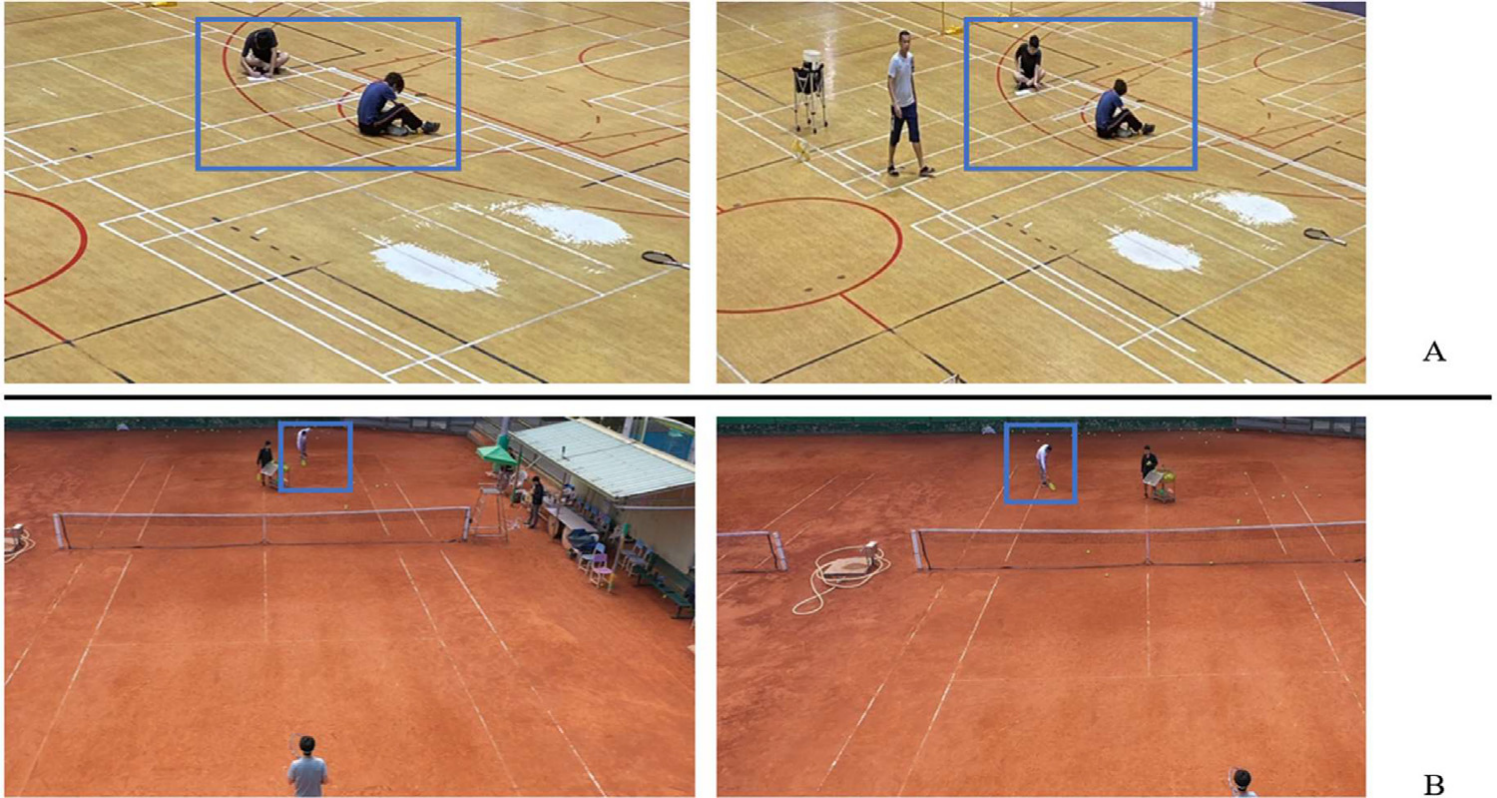
Measurement and recording of data (A) Measurement and recording of the Outdoor Field (Inside the blue box are the surveyor). (B) Measurement and recording of the Indoor field (Inside the blue box are the surveyor).

The landing position of the ball after the flight (the landing position of each ball) is mentioned in the data description. What we do in Outdoor field is to circle the landing position of each ball, number each ball and finally measure and record the actual position. In Indoor field, the landing position of each ball is not marked, so we put a label with a number on it every time the ball hits the ground, and then measure and record the actual position of the ball. The measurement and recording of the data are shown in Fig. 5.

## Limitations

The sources for this article are red clay tennis courts and indoor virtual tennis courts; there are no other sources for material tennis courts. In the future, more tennis courts of different materials will be planned to collect data and interpret tennis ball tracking information on a larger scale.

## Ethics Statement

Before the performance of the experiment, all participants involved in the clip recording provided some data related to physical status and habits of individual, and they read and signed an informed consent form, conserved at Physical Education Office at “National Kaohsiung University of Science and Technology” (the correspondent's office).

## CRediT authorship contribution statement

**Kalin Guanlun Lai:** Conceptualization, Methodology, Software, Writing – original draft, Investigation. **Hsu-Chun Huang:** Validation, Writing – review & editing. **Wei-Ting Lin:** Supervision, Validation, Writing – review & editing. **Shang-Yi Lin:** Validation, Writing – review & editing. **Kawuu Weicheng Lin:** Validation, Writing – review & editing.

## Data Availability

Tennis Shot Side-View and Top-View Data Set for 3D Reconstruction and Player Analysis in Tennis (Original data) (Mendeley Data).
